# CMV neuroretinitis in an immunocompetent patient: a unique case report

**DOI:** 10.22336/rjo.2024.30

**Published:** 2024

**Authors:** Mirza Mariyam Beg, Santosh Kumar, Apurva Bagla, Vinod Kumar Singh, Sonam Verma, Geetanjali Chaparia, Basant Kumar Singh

**Affiliations:** * Department of Ophthalmology, Moti Lal Nehru Medical College, Prayagraj, India

**Keywords:** cytomegalovirus, neuroretinitis, immunocompetent, valganciclovir, ophthalmology

## Abstract

**Aim:** To report a case of cytomegalovirus (CMV) neuroretinitis observed in an immunocompetent patient.

**Materials and methods:** The patient presented with a complaint of diminution of vision in both eyes (BE) and had a traumatic cataract in the right eye (RE). Fundus examination of the left eye (LE) revealed an active white, fluffy lesion with an overlying retinal hemorrhage patch with a macular star. The diagnosis of CMV neuroretinitis was established, and the patient commenced treatment with valganciclovir.

**Results:** The patient exhibited no underlying risk factors. Subsequently, a positive response to oral valganciclovir treatment was observed.

**Discussion:** Cytomegalovirus (CMV) neuroretinitis is typically associated with immunocompromised individuals, such as those with HIV/AIDS. The patient’s presentation with a traumatic cataract in the right eye and a distinctive fundus appearance in the left eye posed a diagnostic challenge. The absence of common risk factors for CMV infection necessitated a thorough examination and consideration of rare infectious etiologies. The positive response to valganciclovir reinforces its efficacy in managing CMV-related ocular conditions. This case emphasized the necessity for ophthalmologists to maintain a high index of suspicion for CMV and other unusual pathogens when faced with neuroretinitis in patients who do not present with typical systemic immunosuppressive conditions. Early diagnosis and appropriate antiviral therapy prevent potential complications and preserve vision in such atypical presentations.

**Conclusion:** This case underscores the importance of considering rare infectious agents in immunocompetent patients when encountering neuroretinitis, particularly in the absence of typical symptoms or signs of the disease.

**Abbreviations:** CMV = Cytomegalovirus, BE = Both eyes, RE = Right eye, LE = Left eye, CBC = Complete Blood Count, ESR = Erythrocyte Sedimentation Rate, VDRL = Venereal Disease Research Laboratory, FTA-ABS = Fluorescent Treponemal Antibody Absorption, PPD = Purified Protein Derivative, ANA = Anti-Nuclear Antibodies, RF = Rheumatoid Factor, ACE = Anti Converting Enzyme, Ig G = Immunoglobulin G, HSV = Herpes simplex virus

## Introduction

Cytomegalovirus (CMV) retinitis stands as the most prevalent intraocular infection observed in individuals with Acquired Immunodeficiency Syndrome (AIDS), affecting roughly 30% of AIDS patients. If left untreated, CMV retinitis progresses relentlessly, spreading across the retina, ultimately leading to complete retinal devastation and ensuing blindness. Traditional systemic administration of antiviral agents like ganciclovir, foscarnet, or cidofovir initially controls the retinitis. However, the propensity for relapse remains relatively high, primarily due to the inadequate ocular bioavailability associated with most compounds administered systemically [**1**].

In this context, valganciclovir emerges as a significant innovation. It is a monovalent ester prodrug that, upon oral administration, promptly converts to its active form, ganciclovir [**2**]. Notably, valganciclovir offers an absolute bioavailability of 60% [**3**], a substantial improvement compared to other orally administered compounds. A dose of 900 mg (equivalent to two 450 mg tablets) yields ganciclovir blood levels akin to those achieved through an intravenous administration of 5 mg of ganciclovir per kilogram of body weight [**4**]. This enhanced bioavailability potentially translates into more effective and sustained therapeutic levels within the ocular tissues, presenting a promising avenue in the management of CMV retinitis in immunocompromised individuals, and potentially extending its utility to rare instances of CMV neuroretinitis in immunocompetent patients.

## Case report

A 40-year-old man came with the main concern of experiencing diminished vision in his right eye, which was more pronounced than in his left eye, persisting for five months. He presented a history of trauma to RE 5 months back. On examination, his visual acuity RE was hand movement, and LE vision was 6/60, with sluggish pupillary reaction in LE. On a slit lamp, he had traumatic cataract RE.

The RE fundus could not be examined on indirect ophthalmoscopy due to cataracts; a normal B-scan was done. Cataract surgery was done in the RE.

**Fig. 1:**
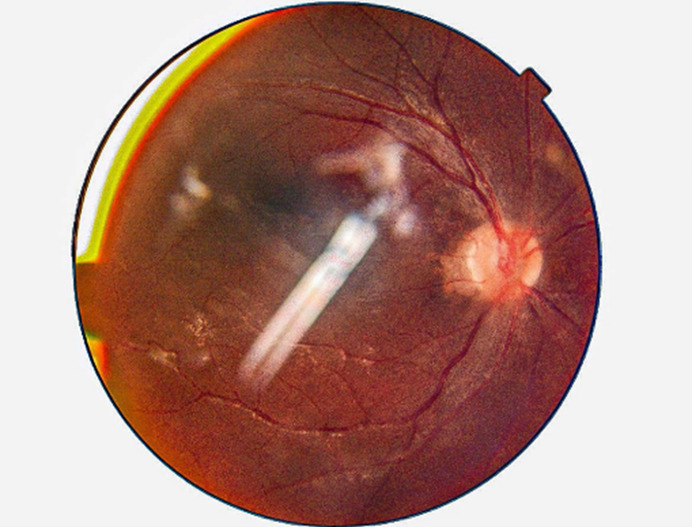
Fundus photo (OD) - within normal limits (OD-Oculus Dexter)

**Fig. 2:**
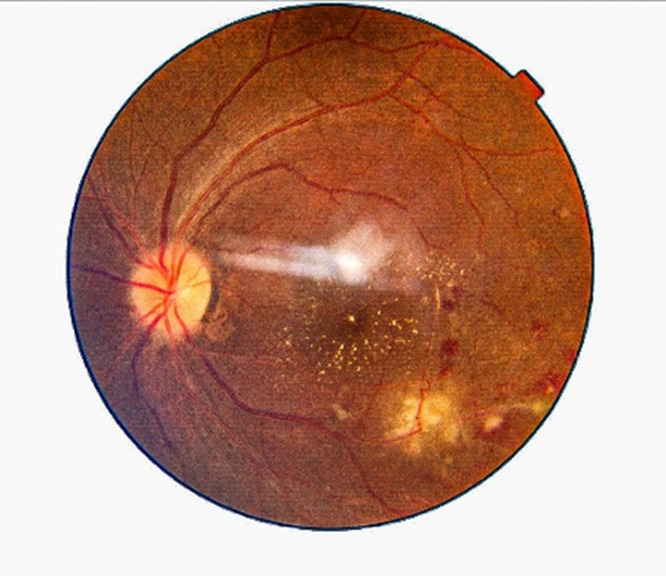
Fundus photo (OS) - pre-treatment - One large approximately half-a-disc diameter white, fluffy lesion with overlying retinal hemorrhages and few scattered necrotic spots along the inferotemporal arcade. Mild optic disc pallor is noted with a macular star about 2DD away from the optic disc (OS-oculus sinister)

**Fig. 3:**
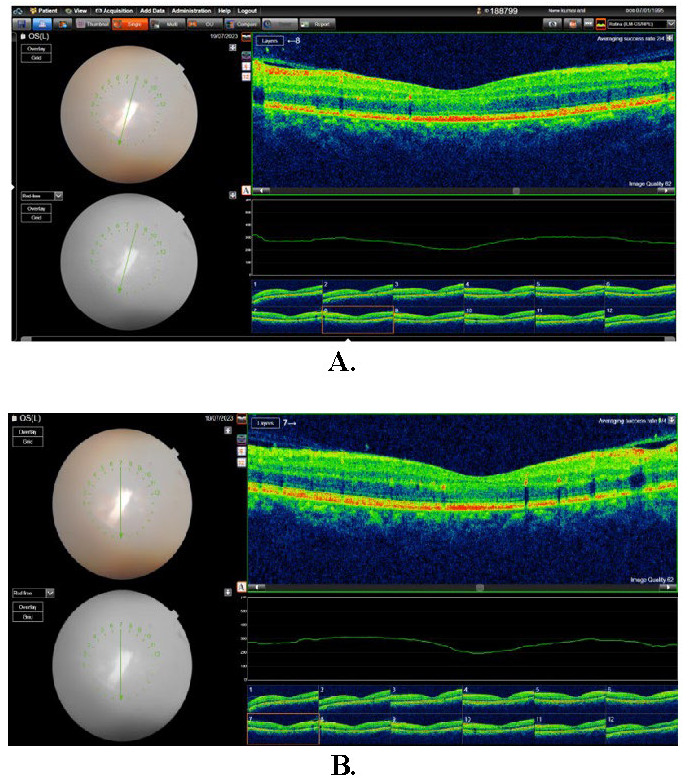
OCT macula LE

**Fig. 4:**
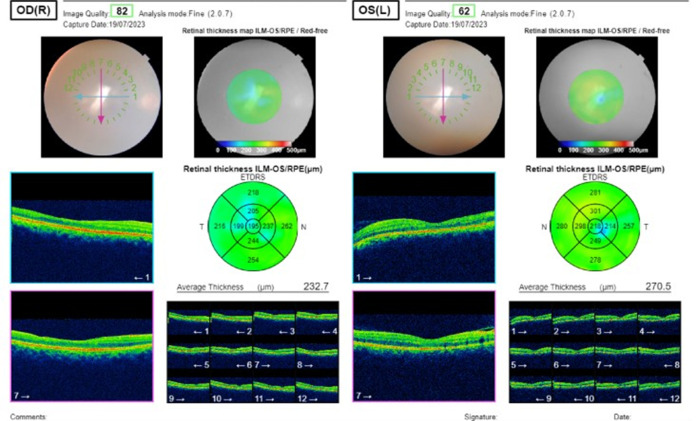
OCT macula - both eyes show macular thinning

Postoperative fundus examination of the RE was within normal limits (**Fig. 1**). At the same time, LE Fundus examination showed one large approximately half-a-disc diameter white, fluffy lesion with overlying retinal hemorrhages and a few scattered necrotic spots along the inferotemporal arcade. Mild optic disc pallor was noted with a macular star about 2DD away from the optic disc, the rest of the fundus was normal (**Fig. 2**). A slit lamp examination showed grade 1 optic disc edema. OCT showed hyperreflective signals at the level of the outer plexiform layer representing retinal exudate (**Fig. 3**, **4**). The remainder of the ocular assessment was within normal limits. The patient did not report any accompanying systemic symptoms, and overall physical examination yielded no abnormalities. All necessary laboratory tests, including Complete Blood Count (CBC), Erythrocyte Sedimentation Rate (ESR), Angiotensin Converting Enzyme (ACE), Fluorescent Treponemal Antibody Absorption (FTA-ABS), Purified Protein Derivative (PPD), Venereal Disease Research Laboratory (VDRL), Antiphospholipid antibody, Anti-Nuclear Antibodies (ANA), Rheumatoid Factor (RF), chest X-ray, anti-HIV antibody, anti-HCV antibody, and anti-HbsAg antibody, were conducted, and the results were within normal ranges except TORCH profile, which showed immunoglobulin G (Ig G) titer of Toxoplasma gondii - 36.785 IU/ml, Rubella virus - 142.690 IU/ml, Cytomegalovirus (CMV) - 1073 AU/ml, Herpes simplex virus (HSV) - 57.335 AU/ml. Immunoglobulin M (Ig M) titers were normal [**5**]. The CD4 cell count of the patient was within the normal range, measuring 950 cells/mm3.

**Fig. 5:**
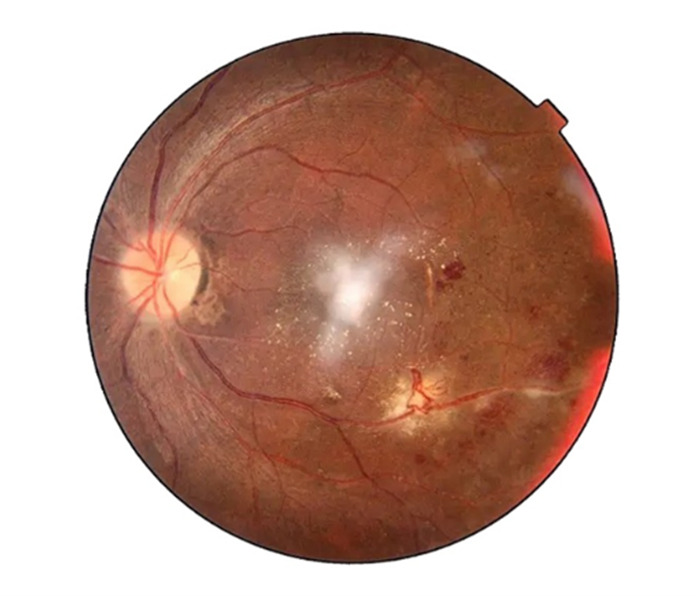
1.5 months after the initiation of treatment

Initially, the treatment regimen included an oral tablet of prednisolone 60 mg/day and a tablet of Septran DS twice daily. Still, the patient did not show any improvement so he was started on valganciclovir - an oral loading dose of Valganciclovir 900 mg once daily dose for 21 days followed by a maintenance dose of 450 mg once daily for 6 weeks and oral prednisolone, which was tapered over 6 weeks. 1.5 months after the initiation of valganciclovir therapy, the patient’s vision improved to 6/6 and the fundus examination showed a healing choroiditis patch with a resolving hemorrhagic patch and macular star (**Fig. 5**). Blood serology (TORCH profile) repeated after 1.5 months showed IgG titer of Toxoplasma gondii - 30.120 IU/ml, Rubella virus - 109 IU/ml, Cytomegalovirus (CMV) - 159 AU/ml, Herpes simplex virus (HSV) - 3.5 AU/ml.

## Results

The patient showed significant improvement in visual acuity and fundus appearance following treatment with valganciclovir. Visual acuity in the LE improved to 6/6, and the fundus lesions resolved, indicating a positive therapeutic response. Repeated serological tests confirmed a reduction in IgG titers for the infectious agents.

## Discussion

CMV progression is promoted by retinal vascular endothelial damage and reduced blood flow velocities associated with HIV infection [**6**]. Loss of T-cell-mediated immune control or changes in the differentiation or activation state of cells harboring latent CMV can result in the reactivation of latent virus and production of viral progeny [**7**].

In immune-competent individuals, mild systemic signs or symptoms related to neuroretinitis that have responded well to antiviral agents are sporadically documented in the literature. For example, a previously published case reported a 28-year-old male exhibiting signs of neuroretinitis alongside a varicella-zoster virus (VZV) IgG-positive status. The patient’s condition resolved completely with oral acyclovir treatment though no polymerase chain reaction (PCR) analysis was conducted [**8**]. Similarly, successful management of neuroretinitis was observed in a 48-year-old female who tested positive for cytomegalovirus (CMV) and herpes simplex virus type 1 (HSV-1) via PCR and IgG assays. Even though with a history of flu-like symptoms, this patient responded well to treatment involving intravenous and intravitreal ganciclovir [**9**].

Neuroretinitis cases have demonstrated associations with various infectious agents. It is crucial to consider rare pathogens were eliminated in cases with more common motifs such as Bartonella henselae [**10**]. Biological examinations, such as specific antibody blood tests or, nucleic acid amplification tests could provide valuable diagnostic insights. PCR testing is an important tool in confirming a diagnosis. However, the reliability of PCR testing on ocular fluids tends to be lower in immunocompetent individuals and not in immunosuppressed patients. In such cases, a negative PCR result cannot rule out the infection, but a positive one can significantly support a diagnosis.

Unfortunately, a PCR test during the acute stage was not conducted in our patient’s case. Therefore, we relied on positive IgG titers for diagnosis. The patient’s favorable response to antiviral treatment and subsequent improvement in serology titers further reinforced this diagnostic approach.

## Conclusion

In conclusion, we documented a case of cytomegalovirus retinitis with a macular star in an immunocompetent individual. It emphasized the significance of considering rare infectious agents in immunocompetent patients, even in the absence of typical symptoms or signs suggestive of the disease.

This underscores the complexity of diagnosing neuroretinitis and the importance of a thorough differential diagnosis approach.

**Conflict of Interest**

The authors state that they do not have any conflicts of interest. 

**Informed Consent and Human and Animal Rights Statements**

Written informed consent has been obtained from the individuals involved in the study.

**Authorization for the use of human subjects**

Ethical approval: The research related to human use complies with all the relevant national regulations, and institutional policies, as per the tenets of the Helsinki Declaration, and has been approved by the review board of Moti Lal Nehru Medical College, Prayagraj, India (ECR/922/inst/UP/RR22 and date of the approval 30 June 2023).

**Acknowledgments**

None.

**Sources of Funding**

The authors received no financial support for the research, authorship, and/or publication of this article. 

**Disclosures**

None.
